# Cell-surface translational dynamics of nicotinic acetylcholine receptors

**DOI:** 10.3389/fnsyn.2014.00025

**Published:** 2014-11-04

**Authors:** Francisco J. Barrantes

**Affiliations:** Laboratory of Molecular Neurobiology, Institute of Biomedical Research, Faculty of Medical Sciences, Pontifical Catholic University of Argentina-National Scientific and Technical Research CouncilBuenos Aires, Argentina

**Keywords:** acetylcholine receptor, cholesterol, lateral mobility, receptor clustering, membrane domains, 2D-diffusion

## Abstract

Synapse efficacy heavily relies on the number of neurotransmitter receptors available at a given time. In addition to the equilibrium between the biosynthetic production, exocytic delivery and recycling of receptors on the one hand, and the endocytic internalization on the other, lateral diffusion and clustering of receptors at the cell membrane play key roles in determining the amount of active receptors at the synapse. Mobile receptors traffic between reservoir compartments and the synapse by thermally driven Brownian motion, and become immobilized at the peri-synaptic region or the synapse by: (a) clustering mediated by homotropic inter-molecular receptor–receptor associations; (b) heterotropic associations with non-receptor scaffolding proteins or the subjacent cytoskeletal meshwork, leading to diffusional “trapping,” and (c) protein-lipid interactions, particularly with the neutral lipid cholesterol. This review assesses the contribution of some of these mechanisms to the supramolecular organization and dynamics of the paradigm neurotransmitter receptor of muscle and neuronal cells -the nicotinic acetylcholine receptor (nAChR). Currently available information stemming from various complementary biophysical techniques commonly used to interrogate the dynamics of cell-surface components is critically discussed. The translational mobility of nAChRs at the cell surface differs between muscle and neuronal receptors in terms of diffusion coefficients and residence intervals at the synapse, which cover an ample range of time regimes. A peculiar feature of brain α7 nAChR is its ability to spend much of its time confined peri-synaptically, vicinal to glutamatergic (excitatory) and GABAergic (inhibitory) synapses. An important function of the α7 nAChR may thus be visiting the territories of other neurotransmitter receptors, differentially regulating the dynamic equilibrium between excitation and inhibition, depending on its residence time in each domain.

## INTRODUCTION

The nAChR abbreviationαBTX, α-bungarotoxin; CDx, methyl-β-cyclodextrin; FCS, fluorescence correlation spectroscopy; FRAP, fluorescence recovery after photobleaching; MSD, mean square displacement; nAChR, nicotinic acetylcholine receptor; SPT, single particle tracking; TIRF, total internal reflection fluorescence.is the prototype of the family of Cys-loop receptors (Nys et al., 2013). This family belongs in turn to the superfamily of ligand-gated ion channels (LGICs), a collection of three evolutionarily unrelated families which include, in addition to the aforementioned Cys-loop receptors, the ionotropic glutamate receptors and ATP-gated channels. The Cys-loop family of pentameric proteins is composed of neurotransmitter receptors with associated anion-selective channels [the γ-amino butyric acid type A (GABA_A_), γ-amino butyric acid type C (GABA_C_), and the glycine receptor] and cation-selective members such as the 5-HT3 (serotonin) receptor and the nAChR (Nys et al., 2013). nAChRs are composed of five polypeptide subunits organized pseudo-symmetrically around a central pore. Each subunit contains an extracellular domain, four hydrophobic transmembrane segments arranged in the form of three concentric rings around the pore (Barrantes, 2003) and a short extracellular carboxy-terminal domain (Karlin, 2002).

In the peripheral nervous system, at the neuromuscular junction in adult myotubes, the receptor macromolecule is highly concentrated in a relatively small area of the cell, juxtaposed and restricted to the endplate, packed at the very high density of 10,000–20,000 particles μm^-2^. Receptor density drops abruptly in the rest of the plasma membrane (<100 particles μm^-2^ Barrantes, 1979; Sanes and Lichtman, 2001). The functional efficacy of the neuromuscular junction, as well as other synapses, heavily depends on its strength. This in turn is directly related to the number of receptors present at the synapse, which depends on the equilibrium between two sets of factors: (i) lateral diffusion into and out of the synaptic region from non-synaptic (“extrasynaptic”) areas, and (ii) the trafficking and turnover of receptors at the cell surface, determined by the rate and extent of biosynthesis and exocytic delivery to the plasmalemma, plus the contribution of receptor recycling back to the surface, on the one hand, and removal of synaptic receptors by internalization (endocytosis) or 2-dimensional diffusion driving them away from the synaptic region, on the other. The latter phenomenon is uncommon in the peripheral synapse. The density of nAChRs at the synapse is also a consequence of the dynamic equilibrium between all these factors (Akaaboune et al., 1999; Bruneau and Akaaboune, 2006). Diffusion into the endplate region is also rare except for accidental or man-tailored conditions such as in denervation hypersensitivity, in which migration of extrasynaptic receptors to the motor endplate occurs in a transient fashion. Several pathological conditions of the neuromuscular junction are associated with an insufficient number of receptor molecules, myasthenia gravis probably being the most prominent example.

In central nervous system (CNS) synapses, the rapid lateral exchange of receptors at the synapse with those in non-synaptic areas is thought to underlie the plastic behavior of excitatory glutamatergic synapses (i.e., those operating through AMPA and NMDA receptors; Choquet and Triller, 2003, 2013; Holcman and Triller, 2006; Triller and Choquet, 2008). Indeed, this dynamic trafficking, and the resulting effective residence time of excitatory synaptic receptors in the active region, directly affects synaptic efficacy and plasticity, that is, long-term potentiation (LTP), long-term depression (LTD) and other biologically important phenomena which lie at the roots of key cognitive functions. GABAergic and glycinergic receptors at inhibitory synapses are dynamically regulated in a similar fashion. As reviewed in this paper, the 2-D translational mobility of nAChRs may impinge on these important processes. α7 nAChRs reside for distinct periods in the neighborhood of glutamatergic and GABAergic synapses, and due to their high Ca^2+^ permeability, are able to differentially regulate the excitatory/inhibitory balance, LTP, and, indirectly, may influence important cognitive functions like learning and memory.

Several neurological and neuropsychiatric disorders have been claimed to be associated with dysfunction of receptors and ion channels, whose alterations are encompassed under the term “synaptopathies.” Diseases like depression, anxiety disorders, various forms of dementia, epilepsy, Parkinson’s disease, autism spectrum disorder, migraine, fragile X syndrome, and schizophrenia are among these disorders, which cover a wide spectrum of pathological synaptic phenotypes, ranging from alterations in the number, size or morphology of dendritic spines, disposition of spines along the dendritic arborizations, etc. The related alterations in these synaptopathies (either hypo- or hyper-function of the synapse) are assumed to depend in turn on the underlying dysfunction of the receptors and channels, the so-called channelopathies (Kass, 2005), which should now be extended to encompass scaffolding and other non-receptor proteins, e.g., those misfolded and aggregated at the synapse, like in Alzheimer’s, Huntington’s or Parkinson’s diseases (for a recent review see, e.g., Remmers et al., 2014).

## ASSESSING THE MOTION OF PROTEINS IN MEMBRANES

The motion of proteins in membranes depends on a multiplicity of factors: the physicochemical properties of the host lipid bilayer, homotropic intermolecular associations of the protein in question (which may or may not be associated with aggregation or clustering), heterotropic association with other proteins (e.g., scaffolding, cytoskeletal, or motor proteins), or lipids, etc. Physicochemical properties of the lipid bilayer (e.g., viscosity) vary from cell to cell and between different membrane compartments in the same cell, but not to the extent that they become a determining factor in protein motion. By far the most important element that influences diffusion in the 2-D plane of the membrane is the degree of association with partner molecules (crowding and clustering), scaffolding proteins or cytoskeletal barriers (corrals), or tethering to the cytoskeleton (Kusumi et al., 1993, 2005; Suzuki et al., 2005; Chen et al., 2006; Jacobson et al., 2007) or lipid platforms (Varma and Mayor, 1998, and see review in Rao and Mayor, 2004, 2014).

Assessing the motion of proteins and lipid in membranes has essentially relied on three complementary techniques: FRAP, FCS, and SPT. For a comprehensive review of the introduction and evolution of these techniques (see, e.g., Day and Kenworthy, 2009). Briefly, FRAP consists of bleaching an area of the membrane containing the fluorescently labeled proteins or lipids in questions with a rapid and relatively intense pulse of light, and then following the time-dependent recovery of the fluorescence signal with a much lower illumination power. The replenishment of the fluorescence signal arises from the diffusion into the photobleached area of fluorescence molecules originally located outside this area. The fluorescence recovery curves are typically characterized by two parameters, a diffusion coefficient (*D*) and a mobile fraction (*Mf*). FCS is also an ensemble method enabling one to study the dynamics (diffusion coefficient), concentrations and molecular interactions (molecular aggregation, binding-unbinding, co-diffusion of two molecular entities, etc.) with high temporal and spatial resolution by following the passage of fluorescently labeled molecules through very small volumes of the cell and analyzing the statistics of fluorescence intensity fluctuations as a function of time (see review in Kim and Schwille, 2003). Recently, the combined application of FCS and superresolution optical microscopy (see section below) has enabled the observation of some of the above phenomena down to the nanometer scale (see, recent review in Eggeling et al., 2013).

Single particle tracking can interrogate the motion of membrane proteins in the native membrane milieu of a living cell by following multiple trajectories of a sufficiently large number of single (e.g., fluorescently labeled) molecules and extracting the apparent average diffusion coefficient from the MSD of the molecules. Some shortcomings of these techniques have been pointed out, such as the invasive nature of FRAP, the essentially “local” interrogation of FCS, and the need to observe isolated particles for relatively long periods of time of SPT (Digman and Gratton, 2009). The limited spatial and/or temporal resolution of these techniques is still subject to criticism, since they provide a “global” or “macroscopic” diffusion coefficient which reflects the overall mobility over areas of several square microns (Sahl et al., 2014). In spite of these criticisms, SPT (Simson et al., 1995, 1998; Saxton and Jacobson, 1997; Dietrich et al., 2002; Chen et al., 2006) still remains the most common approach for analyzing molecular diffusion in membranes, followed by the FRAP technique (see, e.g., Kapitza et al., 1985; Ladha et al., 1994; Niv et al., 2002; Pucadyil et al., 2007). New analytical tools have appeared in recent years to extend the applicability of SPT analysis to more “real life” (e.g., crowding, anomalous diffusion), complicated membrane environments. One such approach is based on Bayesian and Akaike information criteria in information theory for classifying molecular trajectories (Monnier et al., 2012; Türkcan et al., 2012; Türkcan and Masson, 2013; Masson et al., 2014). The Bayesian method has also been combined with superresolution microscopy techniques such as STED to improve the determination of still positions in sub-diffraction images of GPI-anchored membrane proteins (Manzo et al., 2014). The reader is referred to a recent paper (Chenouard et al., 2014), resulting from a competition in which 14 available SPT analytical methods were compared on the same, complex data set – an interesting experiment with no winners, but useful conclusions on applicability.

## LATERAL MOBILITY OF DEVELOPING AND ADULT MUSCLE-TYPE nAChR: FRAP STUDIES

The pioneer study of Axelrod et al. (1976) using the FRAP technique demonstrated that in developing muscle cells the highly clustered nAChRs present in large (20–60 μm) patches are practically immobile, with an effective lateral diffusion coefficient (*D*) of <10^-12^ cm^2^ s^-1^ (<10^-4^ μm^2^ s^-1^). The translational mobility of diffusely distributed nAChRs in other regions of the same plasma membrane is only slightly faster (*D* ∼0.5 × 10^-2^ μm^2^ s^-1^; see **Table 1**).

**Table 1:** Diffusion coefficients of muscle-type nAChR measured by the FRAP technique.

Condition	*Mf*	*D* (μm^2^ s^-1^)	Reference
**Muscle cells**
Developing myotubes, synaptic		<10^-4^	Axelrod et al. (1976)
Extrasynaptic		0.5 × 10^-2^	Axelrod et al. (1976)
Adult rat muscle fibers culture		0.25 × 10^-2^	Stya and Axelrod (1983)
**CHO-K1/A5 cells labeled with Alexa^**488**^-**α***-BTX***
Control cells	0.56 ± 0.09	0.46 ± 0.09 × 10^-2^	Baier et al. (2010)
10 mM CDx-treated	*0.19 ± 0.12	*0.27 ± 0.08 × 10^-2^	Baier et al. (2010)
20 mM Latrunculin A	0.44 ± 0.04	0.67 ± 0.18 × 10^-2^	Baier et al. (2010)
10 mM CDx + 20 mM Latrunculin A	0.28 ± 0.10	0.49 ± 0.21 × 10^-2^	Baier et al. (2010)
3.5 mM CDx-cholesterol (6:1)	0.62 ± 0.08	0.63 ± 0.26 × 10^-2^	Baier et al. (2010)
10 mM CDx-cholesterol (6:1)	0.54 ± 0.10	*1.17 ± 0.44 × 10^-2^	Baier et al. (2010)

CDx, methyl-β-cyclodextrin. Asterisk denotes statistically significant differences, *p* < 0.001.

*From Baier et al. (2010).

The relative immobility of synaptic nAChRs at the neuromuscular junction is probably due to a multiplicity of factors. The muscle endplate and the electromotor synapse of electric fish are compact “islands” with a huge absolute number of receptor macromolecules densely packed at an extraordinarily high density. It is thus not surprising that receptors hardly diffuse in the plane of the membrane… In order to dissect the contribution of intrinsic (e.g., receptor–receptor interactions, clearly apparent, e.g., in early electron micrographs of the *Torpedo* electroplax postsynaptic membrane Heuser and Salpeter, 1979) and extrinsic (e.g., corralling by the submembrane cytoskeletal meshwork) protein clustering factors it is useful to resort to simpler model systems. Heterologous constitutive expression of receptors in cells is a compromise system offering the possibility to conduct a variety of studies under physiological conditions. The clonal cell line CHO-K1/A5 (Roccamo et al., 1999) robustly expresses adult muscle-type nAChR at densities lower than those of the endplate in an adult muscle cell or the motor plate in the electric fish synapses. Recycling of nAChRs is too slow to contribute to the cell-surface pool within the experimentally observed period (Kumari et al., 2008). Furthermore, since one has the possibility to increase the complexity of the model system one building block at a time, the lack of non-receptor scaffolding proteins like rapsyn or the clustering factor agrin make the CHO-K1/A5 a useful mammalian expression system to explore “intrinsic” factors involved in clustering and 2-D diffusion of the nAChR protein and to interrogate in a systematic manner for possible involvement of additional components.

Initial attempts to measure the 2-D mobility of the nAChR at the plasma membrane of CHO-K1/A5 cells and its dependence on membrane cholesterol levels were undertaken using the FRAP technique in the confocal mode (as in Zaal et al., 1999; Nehls et al., 2000). A defined 2-D region was selected from the confocal section of the cell membrane, thus restricting the analysis to a few thousand fluorescent-tagged nAChRs. The region was photobleached by transiently increasing the laser power of the confocal microscope, and the diffusive exchange of bleached proteins with nearby unbleached molecules was then followed using low-intensity laser excitation. Recovery into the bleached region can be described by two parameters, an apparent lateral diffusion coefficient, *D*, and a *Mf* (Edidin, 1994; Chen et al., 2006; Guo et al., 2008). *D* provides a measure of the kinetics of translational mobility, whereas *Mf* reports on the proportion of fluorescent molecules that are able to diffuse back into the bleached area over the time course of the assay (Kenworthy et al., 2004). Using the FRAP technique on αBTX-labeled nAChRs in CHO-K1/A5 cells, we estimated *D* to be 0.46 × 10^-2^ μm^2^ s^-1^ (Baier et al., 2010; **Table 1**), indicating that the lateral diffusion coefficient of the muscle-type nAChR at the cell surface of these cells is quite similar to that of the mobile nAChR fraction in developing rat myotubes (0.5 × 10^-2^ μm^2^ s^-1^; Axelrod et al., 1976) and that of diffusely distributed nAChR in adult rat muscle fibers in cell culture (0.25 x10^-2^ μm^2^ s^-1^; Stya and Axelrod, 1983, 1984; **Table 1**).

## TRANSLATIONAL MOBILITY OF MUSCLE-TYPE nAChR MEASURED BY SPT ANALYSIS

**FIGURE 1:**
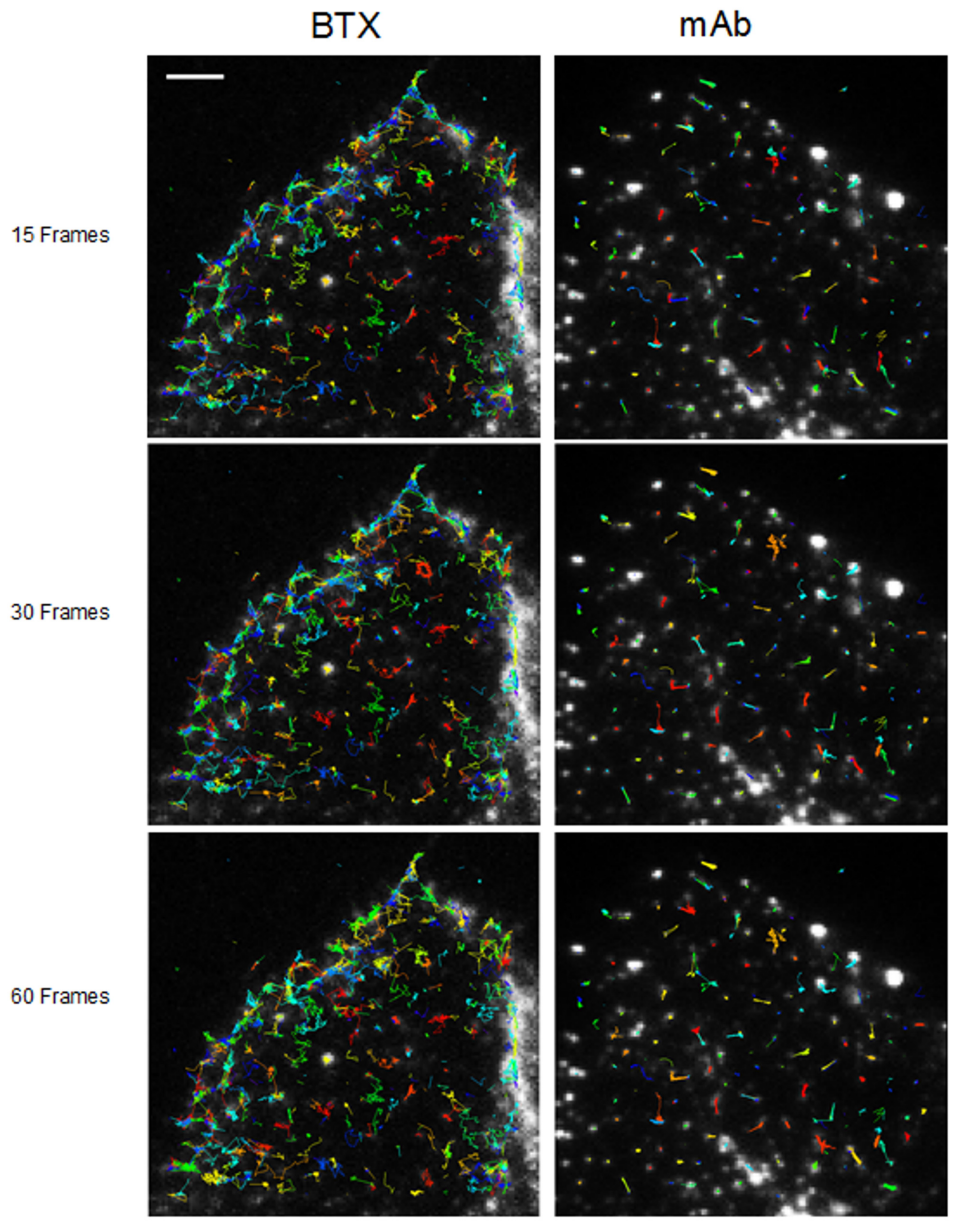
**Multiple trajectories of nAChR particles labeled with BTX and mAb210, respectively.** Sequence of 15 successive frames (out of a total of 1024) corresponding to control BTX- (left column) and mAb (right column)-labeled samples superimposed on the raw TIRF initial frames. Particles were initially localized using a fixed-width Gaussian fitting. Detected particles were subsequently analyzed for their trajectories with the software Localizer (Dedecker et al., 2012) run in an Igor-Pro environment. Typical total number of trajectories was in the order of 800 (4%) and 700 (ca. 10%) out of a total of 15,000 and 8,000 for BTX and mAb-labeled samples, respectively. Scale bar = 3 μm. From Almarza et al. (2014).

Fluorescent-labeled (AlexaFluor^488^α-BTX) nAChR particles imaged with TIRF are diffraction-limited (Borroni et al., 2007; Kellner et al., 2007); yet time-series of up to a few thousand frames are amenable to SPT analysis and useful information can be extracted about their translational dynamics. The density of these puncta is high, yet there is enough contrast and their separation suffices to track the trajectories with a good signal-to-noise ratio. Using the SPT strategy of Danuser and co-workers (Jaqaman et al., 2008) all particles contained within multiple frames from selected sub-regions of CHO-K1/A5 cells were detected in time-series for total durations of ∼25–40 s. **Figure 1** shows the trajectories followed by nAChR particles at the surface of CHO-K1/A5 cells labeled with a monovalent ligand (AlexaFluor^488^α-BTX) or a multivalent ligand (anti-nAChR mAb210 monoclonal antibody followed by AlexaFluor^488^-conjugated IgG secondary antibody) at 4°C. The differences between the two sets of experimental conditions are already apparent from visual inspection of the traces. The motional data derived from the analysis (average dwell-time of the particles, length of their trajectories, average velocity, etc.) are listed in **Table 2** (c.f. Almarza et al., 2014). No particles fell within the region established for immobile particles (“stationary” regime). The microscopic apparent diffusion coefficient *D*_2-4_ (Kusumi et al., 1993) of the receptor labeled with the monovalent ligand α-BTX, shifted from a wide distribution spanning from ∼6.7 × 10^-4^ - 1 μm^2^ s^-1^ (∼6.7 × 10^-12^ - 1 × 10^-8^ cm^2^ s^-1^) to a much narrower distribution with an upper limit close to 5.0 × 10^-4^ μm^2^ s^-1^ upon cholesterol depletion (see **Table 2**; c.f. Almarza et al., 2014). As for antibody-labeled samples, the proportion of slow-moving particles was significantly higher, with a net displacement of particle motion toward the immobile confined regime. *D*_2-4_ values as low as ∼3.3 × 10^-5^ μm^2^ s^-1^ (lower limit) to ∼6.7 × 10^-2^ μm^2^ s^-1^ (upper limit) were observed. Control samples labeled with mAb210 already exhibited a substantial proportion (19.4%) of immobilized particles. This proportion dramatically increased upon cholesterol depletion of the cells, especially during the initial 10 min (83.3%). Interestingly, this short exposure to CDx appears to suffice to alter the mobility properties of monoliganded and mAb-crosslinked nAChRs. The percentage of stationary particles fell to 57.1 and 26.7% after 20 and 40 min treatment with CDx, respectively (Almarza et al., 2014). The MSD of nAChR particles is listed in **Table 3**. Recently Simonson et al. (2010) reported a 2-D diffusion coefficient of 0.1 μm^-2^ s^-1^ for α7-5HT3 chimeric nAChRs heterologously expressed in HEK cells.

**Table 2:** Mobility parameters of nAChR particles in samples labeled with Alexa^**488**^α-BTX or with a primary anti-nAChR monoclonal antibody (mAb210) followed by staining with Alexa^**488**^-labeled secondary antibody, with or without treatment with 15 mM methyl-β-cyclodextrin (CDx).

Experiment	Average lifetime (s)	Average displacement (μm)	Average velocity (μm/ms)	Total no. of particles (in all frames)	Total no. compound tracks analyzed
BTX control	4.06 ± 0.78	4.05 ± 0.27	0.0011 ± 0.0002	4535	121
BTX CDx (10 min)	4.11 ± 0.61	4.54 ± 0.36	0.0010 ± 0.0002	3759	101
BTX CDx (15 min)	5.30 ± 0.80	4.42 ± 0.06	0.0009 ± 0.0001	4574	128

mAb control	10.47 ± 0.31^a^	4.36 ± 0.02^a^	0.0004 ± 0.0001	7772	69
mAb CDx (10 min)	11.06 ± 3.11^b^	2.13 ± 0.25^b^*	0.0002 ± 0.0001	5987	53
mAb CDx (20 min)	13.41 ± 1.44^c^	4.76 ± 0.72^c^	0.0005 ± 0.0001	3755	41
mAb CDx (40 min)	19.96 ± 0.68^d^*	5.70 ± 1.18^d^	0.0003 ± 0.0001	4409	29

*Asterisks denote statistically significant differences (ANOVA test, *p* < 0.05).*

*Average lifetime: d exhibited statistically significant difference with a, b, and c.*

Average displacement: b exhibited statistically significant difference with a, c, and d.

*From Almarza et al. (2014).

**Table 3:** Mean square displacement (MSD) of nAChR particles in samples labeled with Alexa^**488**^α-BTX or with anti-nAChR monoclonal antibody (mAb210) followed by staining with Alexa^**488**^-labeled secondary antibody, with or without treatment with 15 mM methyl-β-cyclodextrin (CDx).

Experimental condition	Total no. of particles (in all frames)	No. of frames analyzed to determine trajectory	Total no. compound tracks analyzed	Mean square displacement (μm^2^)
BTX Control	42,625	15	1702	0.0589 ± 0.0016
		30	1530	0.0936 ± 0.0028
		60	1462	0.1550 ± 0.0056
BTX CDx	18,728	15	1572	0.0874 ± 0.0079
		30	1120	0.1597 ± 0.0177
		60	862	0.2255 ± 0.0206

mAb Control	15,297	15	1476	0.0436 ± 0.0032
		30	1126	0.0671 ± 0.0049
		60	861	0.0974 ± 0.0077
mAb CDx	104,227	15	956	0.0242 ± 0.0019
		30	620	0.0388 ± 0.0031
		60	388	0.0668 ± 0.0105

Analyzed with the software Localizer (Dedecker et al., 2012).

*From Almarza et al. (2014).

## LIPID PLATFORMS AND CHOLESTEROL, THE OBLIGATORY PARTNERS OF THE nAChR

Cholesterol is an abundant component in the postsynaptic membrane (Barrantes, 1989) and it has been demonstrated that this lipid is essential for the nAChR, affecting its distribution and several of its functional properties (Barrantes, 2010, 2012). The lateral heterogeneity of lipids in the postsynaptic membranes of *Torpedo* electrocyte was an early biophysical finding: protein-associated lipids were shown to be immobilized with respect to bulk membrane lipid (Marsh and Barrantes, 1978), and subsequent work has shown that cholesterol-like molecules form part of this protein-immobilized pool (Barrantes, 2007). The functional implications of this finding became apparent when it was demonstrated that cholesterol is an essential component for maintaining nAChR agonist-dependent state transitions in the postsynaptic membrane (Criado et al., 1982). It has been proposed that there are two cholesterol populations in nAChR-rich membranes from *Torpedo:* an easily extractable fraction that influences the bulk fluidity of the membrane and a tightly bound receptor-associated fraction (Leibel et al., 1987). The lipid raft hypothesis proposes that specific lipid species self-associate to form microdomains or platforms that can intervene in protein partition, signaling and other functional events that occur in cell membranes (Simons and van Meer, 1988; Simons and Ikonen, 1997). A fraction of nAChRs occurs in raft domains in mammalian cells, as demonstrated *in vitro* and *in vivo* (Bruses et al., 2001; Marchand et al., 2002; Campagna and Fallon, 2006; Stetzkowski-Marden et al., 2006; Willmann et al., 2006; Zhu et al., 2006), although the purified nAChR protein *per se* exhibits no preference for raft domains *in vitro* (Bermudez et al., 2010). It has also been shown that cholesterol plays a key role in the trafficking of the nAChR along the early exocytic (Pediconi et al., 2004) and endocytic (Borroni et al., 2007; Kumari et al., 2008; Borroni and Barrantes, 2011) pathways and also affects nAChR distribution in the plasma membrane (Borroni et al., 2007; Kellner et al., 2007; Almarza et al., 2014).

Congruent with this series of observations on the multiple roles of cholesterol on nAChR structure and function, cholesterol depletion by CDx treatment produces the accelerated internalization of roughly half of the cell-surface nAChRs in the CHO-K1/A5 cell line (Borroni et al., 2007), an effect exactly opposite to that observed with most other membrane proteins (see, e.g., Kenworthy et al., 2004; Day and Kenworthy, 2009).

Cholesterol has multiple functional impacts on nAChRs. Thus, lowering cholesterol was found to affect nAChR channel properties, producing gain-of-function, as measured by mean open time distribution in single-channel patch-clamp recordings, whereas cholesterol enrichment had the opposite effect (Borroni et al., 2007). CDx-mediated depletion of cholesterol produces a reduction in the fraction of mobile nAChRs from 55 to 20% (Baier et al., 2010). Concomitantly, fluorescence recovery of the toxin-labeled receptor observed in FRAP experiments was clearly slower, yielding an apparent diffusion coefficient (2.1 ± 0.7 × 10^-11^ cm^2^ s^-1^) lower than that in control cells (4.4 ± 0.4 × 10^-11^ cm^2^ s^-1^; **Table 1**). Cholesterol enrichment had the opposite effect. This effect is commonly observed with a wide variety of membrane-embedded proteins (see review by Day and Kenworthy, 2009).

A series of recent publications emphasizes the importance of membrane cholesterol in the biogenesis and stability of nAChR clusters *in vivo* and *in vitro*. In muscle cells, cholesterol was found to influence the formation of micron-sized nAChR clusters induced by agrin (Campagna and Fallon, 2006). Signaling via the agrin/MuSK complex and interaction between the receptor and rapsyn appears to involve lipid platforms (Zhu et al., 2006). Using Laurdan two-photon fluorescence microscopy (Stetzkowski-Marden et al., 2006) it was concluded that nAChR clusters reside in ordered membrane domains. Another study (Willmann et al., 2006) proposed that these cholesterol-rich lipid microdomains and Src-family kinases both contribute to stabilizing nAChRs and the postsynaptic apparatus. As mentioned above, in our experimental clonal cell line, CHO-K1/A5, there are no nAChR-clustering proteins such as rapsyn and tyrosine kinases, and therefore homophilic protein–protein interaction, heterophilic protein-lipid interactions, and links with the actin cytoskeleton are more likely candidates for maintaining the nAChR nanocluster assemblies.

## CLUSTERING OF MUSCLE-TYPE nAChR IN CHO-K1/A5 CELLS

The spatial distribution of nAChRs and other neurotransmitter receptors has been the subject of intense research over the last decades, and it is interesting to gain perspective by looking at what we knew on the subject 35-odd years ago (Barrantes, 1979). Today we can learn about the supramolecular organization and dynamics of receptors, in living cells, with sub-diffraction resolution, as analyzed in the following section on CNS receptors.

**FIGURE 2:**
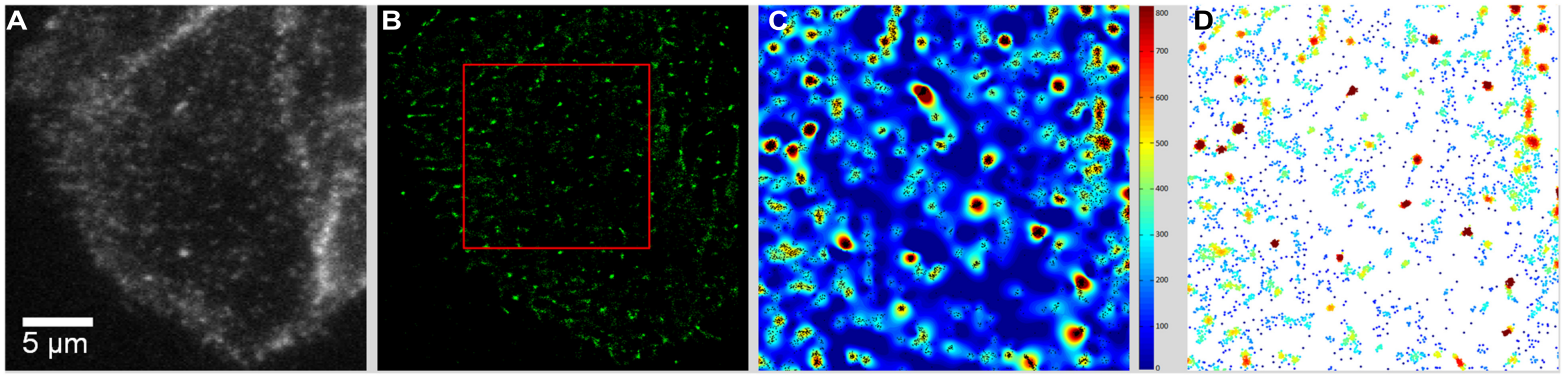
**From raw TIRF images to the graphical rendering of cluster distribution. (A)** TIRF image of CHO-K1/A5 cells stained with Alexa488-α-BTX. The first frame of a movie comprising 1024 frames is shown. **(B)** The output of the QuickPALM reconstruction procedure (Henriques et al., 2010) rendered the totality of particles thresholded above a certain brightness level in the entire movie. The area outlined in red corresponds to a 7.5 μm × 7.5 μm region manually selected for further analysis. **(C)** Cluster map resulting from local-point pattern analysis (Rossy et al., 2013) of the area outlined in red in **(B)**. Visual identification of “hot spots” of clustered particles (black dots) in the entire series of frames. **(D)** Graphical cluster map based on Ripley’s K-function (Owen et al., 2010), pseudo-colored according to relative fluorescence intensity in each individually detected particle. From Almarza et al. (2014).

**FIGURE 3:**
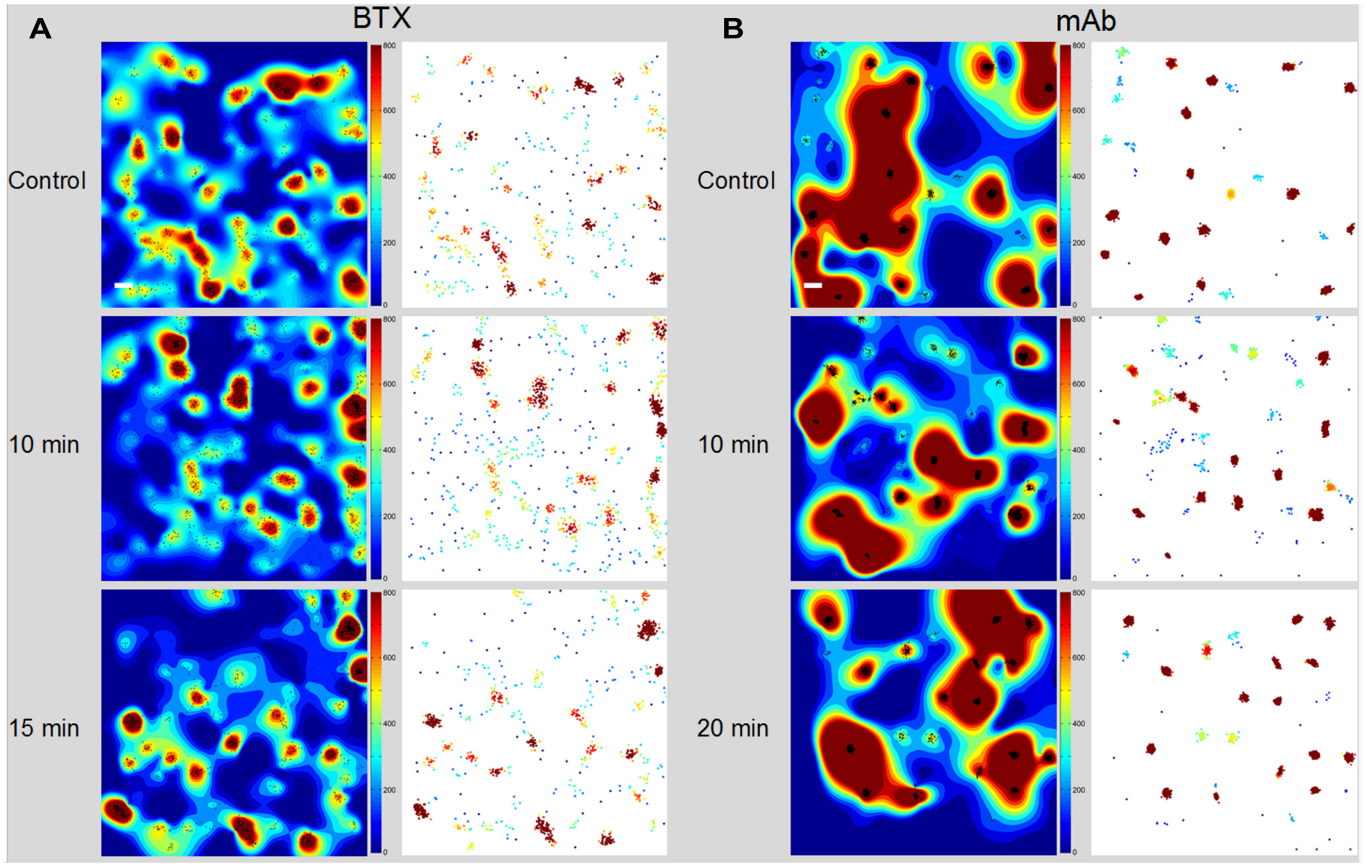
**Time-dependent evolution of the cluster maps. (A)** Alexa488-α-BTX labeled nAChR particles imaged with TIRF microscopy in CHO-K1/A5 cells. The left column shows the interpolated cluster maps resulting from local-point pattern analysis of 4 μm × 4 μm regions in control and CDx-treated cells at the indicated intervals (10, 15 min). The maps, based on Ripley’s K-function (Owen et al., 2010) provide a graphical representation of the degree of aggregation of particles (black dots) per unit area in the entire series of frames. The right column corresponds to the map of clustered BTX-stained particles, pseudo-colored according to relative brightness of the detected particles. **(B)** Time-dependent evolution of the cluster maps of mAb-crosslinked nAChR particles. The left column corresponds to the interpolated cluster map based on Ripley’s K-function applied to CHO-K1/A5 cells labeled with primary anti-nAChR monoclonal antibody (mAb210) followed by staining with Alexa^488^-labeled secondary antibody. The right column shows the map of clustered nAChR particles pseudo-colored according to brightness. Scale bar: 0.2 μm. From Almarza et al. (2014).

TIRF movies of BTX- or antibody-labeled muscle-type nAChR particles recorded from live CHO-K1/A5 were recently analyzed using Ripley’s K-function (Ripley, 1977, 1979) and local point-pattern analysis based on the K-function (Owen et al., 2010; Williamson et al., 2011; Rossy et al., 2013), as shown in **Figure 2**. These methods allow one to examine the spatial organization of the particles by comparing their bi-dimensional point distribution with patterns of complete spatial randomness. Clusters were defined by a maximum nearest neighbor inter-particle radial separation of 200 nm. This dimension is at the limit of the lateral resolution of the TIRF microscopy but is validated -i.e., physically meaningful- by the dimensions of the nAChR nanoclusters resolved by STED microscopy (Kellner et al., 2007). The maps (Owen et al., 2010) graphically categorize areas of particle isodensity; discrete “hot spots” showing the highest degree of particle aggregation can be clearly identified in the two-dimensional projections stemming from the entire series of frames. **Figure 3A** shows the time-dependent evolution of the quantitative cluster maps of BTX-labeled nAChR particles in control and 10 and 15 min after CDx treatment of the cells, respectively. The right column in **Figure 3A** depicts particles sorted according to their relative brightness. The extremely bright clusters identified by this criterion match the clustered regions sorted by positional recognition in the left column of **Figure 3A**. The quantitative local point-pattern analysis based on Ripley’s K-function (Owen et al., 2010; Williamson et al., 2011; Rossy et al., 2013) clearly indicated that nAChR particles were not randomly distributed but organized in clusters, which differed in size, brightness and density between BTX and antibody-treated samples (**Figure 3B**). Their arrangement also changed as a function of time of exposure to CDx, reaching a maximum at 10 min treatment for BTX- and 20 min for mAb-labeled samples (**Figure 3**), in accordance with the kinetics observed in SPT data (**Table 2**).

An additional ensemble analytical tool, the pair correlation function *G(r)* (Greenfield et al., 2009) was applied to the experimental data to measure the properties of individual clusters, averaged over many clusters. Values of *G(r)* > 1 indicate non-random distribution, which can be assumed to be particle clustering (Perry et al., 2006; Kiskowski et al., 2009; Sengupta et al., 2011). Particles exhibited a high degree of clustering at very short–length scales in the control sample as compared to particles in cells treated with CDx, which extended its non-random, clustered pattern up to a radius >1 μm. The statistics of nAChRs distribution between disperse and clustered particles are shown in **Table 4**.

**Table 4:** Distribution of free and clustered nAChR particles in CHO/K1-A5 cells (see also **Figure 3**).

Experiment	Total number of particles	Particles in clusters	
BTX control	938.1 ± 214^a^	895.8 ± 209 (95.1%)^a^	
BTX CDx (10 min)	514.4 ± 192^b^*	470.6 ± 182 (91.4%)^b^*	
BTX CDx (15 min)	931.2 ± 262^c^	886.6 ± 269 (95.2%)^c^	

mAb control	736.7 ± 474^d^*	680.7 ± 455 (92.3%)^d^*	
mAb CDx (10 min)	8930.8 ± 3200^e^*	8859.9 ± 3183 (99.2%)^e^*	
mAb CDx (20 min)	5521.3 ± 2776^f^	5487.3 ± 2761 (99.34%)^f^	
mAb CDx (40 min)	1151.1 ± 995^g^	1134.3 ± 990 (98.54%)^g^	

*Total number of particles: b exhibited statistically significant difference with a and c; d exhibited statistically significant difference with e, f, and g.*

*Particles in clusters: b exhibited statistically significant difference with a and c; d exhibited statistically significant difference with e, f, and g.*

Brightness: a exhibited statistically significant difference with b and c; a exhibited statistically significant difference with b and c.

*From Almarza et al. (2014).

One important piece of information stemming from this and other studies analyzed in this review is the relationship between the motional regimes that receptor molecules undergo and their supramolecular organization, as well as the effect of non-receptor scaffolding proteins on the latter. It has long since been known that rapsyn (formerly called 43K protein) affects nAChR distribution at the cell surface (Barrantes et al., 1980; Burden et al., 1983; Ramarao and Cohen, 1998). This effect varies along development, as illustrated in a recent study on the effect of rapsyn on nAChR mobility followed along myoblast development in culture (Piguet et al., 2011). The myristoylated N-terminus of rapsyn molecules anchors nAChRs to the plasma membrane in a 1:1 stoichiometry, playing a major role during myoblast differentiation and neuromuscular junction development. In myoblasts the majority of the receptors were found to be immobile, with 20% of the receptors exhibiting restricted diffusion in small domains of about 50 nm (Piguet et al., 2011). Before differentiation, only 2% of the nAChRs showed Brownian diffusion, 24% diffused in confined regions, and 74% were immobile. Upon differentiation into multinucleated myoblasts, a strong diminution of the immobile fraction was observed, in conjunction with an increase in the proportion of confined diffusing receptors from 20 to 34%, and Brownian-diffusing receptors from 2 to 10%. In a myoblast cell line devoid of rapsyn, the fraction of mobile nAChRs was higher, and was accompanied by a threefold decrease in the immobile population in comparison to rapsyn-expressing cells. About 50% of the mobile receptors were confined to domains of about 120 nm. Irrespective of the presence of the nAChR-anchoring protein rapsyn, nAChR was confined to domains : when rapsyn was present, the size of the domains diminished (Piguet et al., 2011). This study is in agreement with our study using direct imaging of nAChR nanoclusters using superresolution microscopy in cells devoid of rapsyn (Kellner et al., 2007).

## EFFECT OF CHOLESTEROL ON nAChR TRANSLATIONAL MOBILITY

Several FRAP studies have shown that cholesterol depletion affects the mobility of various proteins at the plasma membrane although the nature, extent and sign of the changes remain a contentious subject. In FRAP experiments performed on cells treated with Mevinolin, a statin that inhibits cholesterol biosynthesis, we found that nAChR mobility was affected in a manner similar to that reported here using CDx mediated acute cholesterol depletion (Baier et al., 2010; **Table 1**). FCS in the confocal microscopy modality corroborated the results of FRAP microscopy. Whereas values of *D* of 5.3 ± 0.4 × 10^-2^ μm^2^ s^-1^ were observed in control cells, D was reduced to 3.7 ± 0.3 × 10^-2^ upon cholesterol depletion (Baier et al., 2010). On the basis of these observations, we can conclude that plasma membrane fluidity is not the main factor determining nAChR mobility.

Some authors reported that the mobility of raft- and non-raft resident proteins decreases when cholesterol is removed from the plasma membrane (Kenworthy et al., 2004; O’Connell and Tamkun, 2005). Restricted diffusion of membrane proteins upon cholesterol depletion is believed to result from the formation of solid-like clusters in the membrane (Vrljic et al., 2005; Nishimura et al., 2006). Sun et al. (2007) postulate that cholesterol affects the mechanical properties of plasma membrane through the underlying cytoskeleton. Using single-molecule tracking methods, another group (Orr et al., 2005) found that cholesterol depletion produces confinement of the epidermal growth factor receptor and human epidermal growth factor receptor 2 mobility, whereas cholesterol enrichment extended the boundaries of the mobility-restricted areas. In contrast, other authors observed an increase in the lateral mobility of the raft-resident proteins CD44 and wild-type GFP-H-Ras after cholesterol depletion (Oliferenko et al., 1999; Niv et al., 2002). Removal of cholesterol, particularly with CDx, not only alters membrane viscosity but can also hinder membrane protein diffusion (Shvartsman et al., 2006).

There is evidence of interactions between lipids, lipid domains and the cytoskeleton (Maxfield, 2002; Lenne et al., 2006; Honigmann et al., 2014). According to Kwik et al. (2003) cholesterol depletion produces general effects on the architecture and function of the membrane, making the sub-membrane cytoskeleton and in particular the cortical actin network more stable. Such a reorganization of the actin meshwork would be associated with reduced receptor mobility. Using FCS and STED it was recently shown that membrane-bound actin networks influence lipid phase separation; a model combining the coupling of membrane composition, membrane curvature, and the actin pinning sites was postulated from this study (Honigmann et al., 2014). More recently, confocal FRAP distinguished two protein populations of membrane proteins, including some classical “synaptic” proteins in PC12 cells, having diffusion coefficients *D* of 0.22 and 0.01 μm^2^ s^-1^, respectively (Saka et al., 2014). When FCS in the superresolution mode (STED-FCS) was applied, the spatio-temporal resolution afforded the determination of *D* on fast diffusing molecules (slowly diffusing or immobile molecules do not traverse the observation spot and do not cause intensity fluctuations, thus precluding their detection). *D* was found to be 0.1–0.6 μm^2^ s^-1^ for the highly mobile protein fraction, which varied inversely proportional to molecular density. Interestingly, cholesterol level was found to be the most important factor in determining protein mobility *and* stabilizing protein assemblies (clusters; Saka et al., 2014).

## DIFFUSIONAL MODULATION AND CONFINEMENT OF nAChR ASSEMBLIES BY CYTOSKELETAL COMPONENTS AND SCAFFOLDING PROTEINS

Cytoskeletal interactions have been shown to modulate the diffusion and confinement of several membrane proteins (Triller and Choquet, 2003; Kusumi et al., 2005; Suzuki et al., 2005). In the case of the muscle-type nAChR, cholesterol depletion affected the long-range relationship of nAChR nano-clusters of ∼55 nm diameter, changing from a random to a non-random distribution (within a radius of 0.5–1.5 μm) upon depletion (Kellner et al., 2007). Interactions of these nano-clusters with the cytoskeleton were invoked as a possible explanation for these changes since nAChR mobility at the plasma membrane appears to be sensitive to the integrity of the cytoskeleton (Stya and Axelrod, 1983; Bloch et al., 1989; Pumplin and Strong, 1989; Dai et al., 2000). Furthermore, interaction between nAChR molecules and the cytoskeleton is of physiological and developmental importance: it is a requisite step in the formation and stability of the neuromuscular junction (Hoch, 1999). In subsequent work from our laboratory the effects of cytoskeleton disruption on nAChR dynamics (Baier et al., 2010) were experimentally explored. Even though cholesterol depletion-induced loss of nAChR mobility was partially restored in cells incubated with Latrunculin A (**Table 1**; Baier et al., 2010), the percentage of mobile nAChRs in these cells did not reach control levels. From this we concluded that although the cortical actin meshwork is likely involved in receptor mobility at the cell surface in cholesterol-depleted cells, it is not necessarily the only factor influencing nAChR translational diffusion. Other cortical cytoskeletal proteins and/or actin-binding proteins may be involved, and direct interactions of cholesterol with the nAChR may also be implicated. Furthermore, inhibition of actin polymerization by cytochalasin *D*, which binds to the barbed end of the actin filament and blocks monomer addition, resulted in inhibition of nAChR internalization (Kumari et al., 2008). However, direct effects of cholesterol on the nAChR cannot be discarded when considering the profound influence of this lipid on the macromolecule’s cell surface mobility.

The nAChR *Mf* may correspond to the nAChR oligomeric forms observed in negatively stained electron micrographs (Barrantes, 1982), which are exchangeable with relatively less mobile nAChR aggregates in larger nano-clusters (Kellner et al., 2007). We have hypothesized that lowering cholesterol levels would affect mostly the more rapidly diffusing nAChRs due to enhanced nAChR–nAChR interactions, which would decrease their residence time at the cell surface (Barrantes, 2007) and result in their internalization (Borroni et al., 2007). The effect of homophilic interactions in membrane proteins was exemplified in an experimental work and model for syntaxin molecules’ self-organization at the plasma membrane (Sieber et al., 2007), in which weak homophilic protein–protein interactions were responsible for syntaxin clustering, syntaxin molecules in these clusters dynamically exchanging with freely diffusing molecules.

In brain, glycine receptors are stabilized by microtubules in extrasynaptic regions, and by gephyrin and actin filaments in synaptic regions (Charrier et al., 2006); AMPA receptors become stable upon interaction with the protein GRIP1, which binds in turn to microfilaments (Allison et al., 1998). Disruption of the cytoskeleton or the microtubule networks with Latrunculin A or nocodazole, respectively, affected the mobility of the neuronal α7 nAChR but not its ability to form clusters, as we have observed in muscle-type nAChRs using superresolution microscopy (Wenz et al., 2010). The exact mechanisms of nAChR immobilization in CNS synapses and in particular the role of the cytoskeleton or other diffusional traps merit further investigation.

Which other factors may contribute to nAChR mobility, trafficking and clustering? Various post-translational modifications are known to occur in nAChRs: the macromolecule is the target of disulphide bond formation, glycosylation, phosphorylation, palmitoylation, and other modifications which might affect nAChR dynamics. Palmitoylation of assembling α7 subunits in the endoplasmic reticulum has been shown to play a role in the formation of functional αBTX sites (Drisdel et al., 2004; Alexander et al., 2010). A linear relationship has been found between average nAChR half-life and the percentage of nAChR with phosphorylated β subunit in cultured muscle cells. Phosphorylation occurs specifically at tyrosine residue 390 of the β subunit, and is induced by agrin. This unexpected role of agrin in downregulating AChR turnover most likely stabilizes nAChRs at developing synapses and contributes to the extended half-life of the receptors at adult NMJs (Rudell and Ferns, 2013). Phosphorylation-induced global conformational changes have been recently proposed to be a universal phenomenon among LGICs, and also to play a role in pathophysiological phenomena such as nicotine addiction in the specific case of the nAChR (Talwar and Lynch, 2014).

## ANTIBODY-MEDIATED nAChR CROSSLINKING RESTRICTS RECEPTOR MOBILITY

Muscle nAChR is the target auto-antigen in the autoimmune disease myasthenia gravis. Neuromuscular dysfunction in this disease is caused primarily by the crosslinking of autoantibodies to the endplate nAChR, although other antigens such as muscle-specific tyrosine kinase and low-density lipoprotein receptor-related protein 4 are currently recognized as molecular targets in muscle (Sieb, 2014). Antibody binding results in impaired receptor function, diminished neuromuscular transmission and clinical symptoms: weakness and rapid-onset fatigue. Antibody binding also triggers the endocytic internalization of nAChRs in C2C12 muscle cells and in CHO-K1/A5 cells (Kumari et al., 2008). Thus, the effect of antibodies on muscle nAChRs is not only of biological but also of medical importance.

In agreement with nAChR crosslinking studies in rat myotubes in primary culture (Axelrod, 1980), antibody-induced crosslinking results in a marked diminution of receptor mobility in adult-type nAChR expressed in CHO-K1/A5 cells. Employing the SPT technique, instead of the long particle walks observed with a monovalent ligand such as α-BTX, the motion of antibody-crosslinked nAChR particles was restricted to much shorter trajectories confined within relatively small areas (**Table 2**; cf. Almarza et al., 2014).

## NEURONAL-TYPE nAChR MOBILITY

The dynamics of neuronal nAChRs have also been studied with biophysical techniques. One preparation that has proved suitable for this type of studies is the mouse submandibular ganglion (McCann et al., 2008). In the synapses between pre- and post-ganglionic neurons in this ganglion, the density of synaptic receptors is normally maintained by the combination of exchange of receptors with non-synaptic regions, a diffusional phenomenon occurring in the time course of minutes, and the turnover of cell surface receptors, taking place in the course of hours. To measure the kinetics of α7 nAChR, McCann et al. (2008) resorted to various techniques. First, using fluorescent α-BTX they identified postsynaptic and non-synaptic populations of nAChRs. Postsynaptic nAChRs remained stable for days; non-synaptic nAChRs were more dynamic, being replaced in the course of days. Secondly, using the FRAP technique the authors studied nAChR lateral diffusion in the ganglionic neurons, measuring a t_1/2_ of recovery of 47 ± 7 min and 11 ± 4 min for synaptic and non-synaptic α7 nAChR clusters, respectively. Thirdly, to measure the turnover rate of nAChRs *in vivo*, McCann et al. (2008) resorted to a fluorescence and pulse-chase technique (Akaaboune et al., 1999) which enabled them to follow the fate of the nAChRs in the living animal for several days. The rate of loss of cell-surface neuronal α7 nAChRs (350 ± 47 min) was found to be 60-fold faster than that of muscle-type nAChRs at the neuromuscular junction (Akaaboune et al., 1999; Bruneau and Akaaboune, 2006). If living ganglion cell axons were severed, synaptic receptors showed enhanced lateral mobility and insertion of new receptors dramatically decreased, leading to near-complete loss of synaptic receptors and to acute synaptic depression. Disappearance of postsynaptic spines and presynaptic terminals ensued (McCann et al., 2008). The authors concluded that rapid changes in synaptic efficacy precede long-lasting structural changes in synaptic connectivity. FRAP continues to be applied to the study of neuronal nAChRs. In a recent study, FRAP revealed that the agonist nicotine, acting on α7 nAChRs in hippocampal postsynaptic neurons, induces the stabilization and accumulation of GluA1-type AMPA receptors (Halff et al., 2014).

In the CNS, the two most abundant forms of nAChR are the heteropentameric oligomer formed by α4 and β2 subunits and the homopentameric receptor formed exclusively by α7 subunits (Gotti et al., 2009). The α7 nAChR is found in the neuronal soma and also pre-, post-and peri-synaptically. Presynaptic α7 nAChRs modulate the release of various neurotransmitters, and postsynaptic α7 nAChRs are involved in the generation of postsynaptic currents (Cuevas and Berg, 1998). Postsynaptic α7 nAChRs can be associated with dendritic spines, in a peri-synaptic annulus (Fabian-Fine et al., 2001). Peri-synaptic α7 nAChRs are found in the vicinity of GABAergic and glutamatergic synapses (see below and, e.g., Buerli et al., 2010). The α7 nAChR exhibits unique functional properties that distinguish it from other nicotinic receptors: (a) fast desensitizing kinetics, (b) unusually high Ca^2+^ permeability, and (c) high affinity for binding αBTX (Alkondon et al., 1997; Alkondon and Albuquerque, 2004). The α7 nAChR is highly expressed in the hippocampus and in GABAergic interneurons in particular. The hippocampus is one of the brain regions mostly affected in Alzheimer disease, where it regulates inhibition of hippocampal networks: activation of α7 nAChR blocks the induction of short-term potentiation as well as LTP. It is involved in cognition and has been associated with pathological states other than Alzheimer disease, such as schizophrenia and Parkinson disease (Banerjee et al., 2000).

In the Introduction I described the mechanisms involved in the maintenance of physiological numbers of receptors at the synapse. In the case of CNS synapses, 2-D diffusion plays an additional role in this equilibrium since receptors need to abandon the postsynaptic region, diffusing away before undergoing endocytosis, a process which appears to occur exclusively in extrasynaptic areas (Blanpied et al., 2002).

In another study of α7 nAChR mobility in cultured hippocampal neurons, SPT was carried out on a small fraction of receptors labeled with quantum dot-coupled α-BTX (Buerli et al., 2010). It should be mentioned that in hippocampal neuronal cultures the GABAergic interneurons are not expected to receive cholinergic innervation, since they are deprived of inputs from distal anatomical brain regions such as the septum. In spite of the absence of synaptic input, α7 nAChRs clusters are present on the neuronal surface. Less than 20% of the receptors were found in clusters, categorized as “synaptic,” as opposed to those labeled with the presynaptic marker synapsin 1, which were assigned to dendritic, postsynaptic, nicotinic sites. The majority (78%) of the receptors were found in the form of aggregates in extrasynaptic areas and were either classified as “axonal” (20%, highly mobile, *D* > 0.1 μm^2^ s^-1^, Brownian motion with mostly linear trajectories) or peri-synaptic, i.e., in the vicinity of, but not colocalized with, excitatory glutamatergic (identified by mCherry-Homer 1c staining) and inhibitory GABAergic (labeled with EGFP-gephyrin) postsynaptic densities. The α7 nAChRs in perisynaptic locations differed in their mobility, too, with lowest receptor mobility (>66% of the peri-GABAergic with *D ∼*0.018 ± 0.03 μm^2^ s^-1^ and >70% of the peri-glutamatergic with *D ∼*0.028 ± 0.04 μm^2^ s^-1^), reflecting local confinement domains, these differences suggesting in turn that the tethering mechanisms holding these nicotinic receptors in the vicinity of excitatory and inhibitory synapses differed as well (Buerli et al., 2010). What are the possible physiological implications of these findings? Stimulation of α7 nAChRs in hippocampal interneurons modulates GABAergic inhibitory postsynaptic potentials, depressing them in some cases (Wanaverbecq et al., 2007) or exciting them in other instances (Ji and Dani, 2000). In the latter case, the ACh-induced excitation of the bicuculline-sensitive GABAergic interneurons could in turn excite or inhibit pyramidal neurons in the CA1 region. Methyllycaconitine-sensitive α7 nAChRs also appear to affect glutamatergic synapses, modulating back-propagating dendritic action potentials and, hence, LTP (Rosza et al., 2008). Activation of (seven AChRs influences postsynaptic NMDA receptors, relieving the Mg2+ block and thus enhancing the probability of LTP induction (Dani and Bertrand, 2007). From this type of evidence, the conclusion was reached that their peri-synaptic localization and their high Ca^2+^ permeability endows α7 nAChRs with the ability to regulate both excitatory and inhibitory CNS synapses independently of their endogenous transmitter (Buerli et al., 2010).

Chick ciliary ganglion neurons in culture express homomeric α7 and heteromeric α3 nAChR at their surface. nAChR lateral mobility was measured using biotinylated α-BTX and biotinylated monoclonal antibody against α3 nAChRs, respectively, followed by streptavidin-coated quantum dots with an emission wavelength of 605 nm (Fernandes et al., 2010). In the case of α3 nAChRs, only 34% were mobile. The resulting diffusion coefficient, *D*, was reported to be 0.070 μm^2^ s^-1^ and 0.188 μm^2^ s^-1^ in synaptic (roughly 50%) and extrasynaptic regions, respectively. In the case of α7 nAChRs the *Mf* was much higher (61%) and the measured *D* was 0.067 and 0.188 μm^2^ s^-1^ for synaptic and extrasynaptic locations, respectively (Fernandes et al., 2010). The dwell time at the synaptic region was about 0.5 ms for the two types of neuronal nAChRs. Analysis of the MSD indicated that synaptic receptors exhibited constrained motion, and extrasynaptic receptors displayed Brownian motion. That is, when either type of receptors is able to diffuse freely, they do so at similar rates, but when their motion is restricted, their constraints differ. In adult ciliary ganglia *in vivo* α7 nAChRs are localized in the peri-synaptic region; in cultured neurons, wide-field microscopy immunocytochemistry showed puncta in close proximity to synaptophysin labeling (Fernandes et al., 2010).

## CHOLESTEROL AND SCAFFOLDING PROTEINS DIFFERENTIALLY AFFECT NEURONAL α3 AND α7 nAChR MOBILITIES

Ciliary ganglion neurons were the first test preparation where α7 nAChRs were reported to occur in lipid “rafts” in somatic spines (Bruses et al., 2001). In their quantum dot SPT study of chick ciliary ganglion neurons, Berg and coworkers (Fernandes et al., 2010) found that α7 and α3 nAChRs had similar mobilities, but differed in the nature of their synaptic restraints. Furthermore, cholesterol depletion by treatment with cholesterol oxidase increased the mobility of extrasynaptic α3 nAChRs from 0.188 to 0.208 μm^2^ s^-1^ without affecting the proportion of immobile α7 nAChRs.

In contrast, cholesterol depletion affected both synaptic and extrasynaptic α7 nAChRs, and the proportion of receptors visiting synaptic territory increased. Cholesterol depletion also raised the proportion of mobile α3 nAChRs from 34 to 54%, without affecting that of α7 nAChRs. Disruption of PDZ-containing scaffolds or of actin filaments in chick ciliary ganglion neurons increased the mobility of α7 nAChRs but not that of α3, as expected from the wealth of evidence on the role of the actin and PDZ-scaffolds in maintaining synapse, and in particular dendritic spine, architecture (Hotulainen and Hoogenraad, 2010). It has been previously reported that in one cell, a single species of protein can have one subset undergoing Brownian diffusion and other subsets undergoing confined or anomalous diffusion (Feder et al., 1996). Muscle-type nAChR mobility also displays a strong dependence on cytoskeletal integrity (Bloch et al., 1989; Pumplin and Strong, 1989; Dai et al., 2000) in developing myotubes and in the adult neuromuscular junction.

## SIMILARITIES AND DIFFERENCES BETWEEN MUSCLE-TYPE AND NEURONAL-TYPE nAChRs: MOTIONAL DYNAMICS AND CLUSTERING ABILITY

Using FRAP and FCS, two ensemble methods suitable for interrogating membrane protein mobility, we found that the mobility of the adult murine muscle-type nAChR heterologously expressed in the clonal cell line CHO-K1/A5 (Roccamo et al., 1999) is dependent on cytoskeletal integrity (Baier et al., 2010). In these cells the nAChR does not form clusters several microns in length as in adult myotubes, but aggregates in the form of very small, nanometer-sized nanoclusters (Kellner et al., 2007), probably because CHO/K1-A5 cells lack rapsyn and other scaffolding or receptor-anchoring proteins like agrin. An equivalent assembly in the CNS cholinergic synapses has not been experimentally demonstrated to date. Our recent SPT study (Almarza et al., 2014) reinforces the conclusion of Berg and coworkers (Fernandes et al., 2010) on the receptor-subtype specificity of the motional regime adopted by different nAChRs. The muscle-type nAChR in CHO-K1/A5 cells is inherently mobile, and only a modest proportion (20%) is immobilized by antibody crosslinking. A dramatic (83%) but transient increase in the percentage of immobile receptors is observed upon cholesterol depletion of the cells, especially during the initial 10 min. The percentage of stationary particles fell thereafter to 57% (20 min) and 27% (40 min) when cells having antibody-crosslinked receptors were additionally depleted of cholesterol. Thus, antibody crosslinking and cholesterol depletion exhibited a synergistic, time-dependent effect (Almarza et al., 2014).

The stability of the adult muscle-type nAChR nanoclusters at the cell surface is modulated by the size of their supramolecular organization – nAChR nanoclusters increase in size upon cholesterol depletion (Kellner et al., 2007) – and hence by the number of receptor units in the nanocluster. Nanoclusters are subsequently internalized (Borroni et al., 2007), further reducing the density of nAChRs at the cell surface. Similarly, in ciliary ganglion neurons cholesterol depletion also reduces the number of α3 nAChRs but not that of α7 nAChRs at the cell surface (Fernandes et al., 2010). Cholesterol, synergistically coupled to other factors determining the size of the nAChR nanoclusters, could thus exert homeostatic control over receptor levels and the dynamics of the nAChR supramolecular assemblies at the cholinergic synapse.

*Caenorhabditis elegans* provides an interesting model system to explore the interplay between neurotransmitter receptors and scaffolding proteins, and to exploit the genetic manipulation of this singular animal to gain insight into the mechanisms involved. At *C. elegans* NMJs, it has been possible to show *in vivo* that extracellular scaffolding proteins are required to cluster the levamisol-sensitive nAChRs (L-nAChRs) in the nematode. The ectodomain of the transmembrane protein LEV-10 and a second extracellular protein, LEV-9, are involved in this process (Gendrel et al., 2009). LEV-9 is a multidomain factor containing complement control protein modules. LEV-9 is secreted by the muscle cells and needs to be proteolytically cleaved at its C terminus to exert its function (Briseño-Roa and Bessereau, 2014).

## MOTION OF OTHER BRAIN NEUROTRANSMITTER RECEPTORS IN CROSSTALK WITH nAChRs

In brain, most neurotransmitter receptors not anchored to diffusional traps or scaffolding domains appear to freely diffuse on the plane of the membrane at rates between 0.1 and 0.5 μm^2^ s^-1^ (Meier et al., 2001; Choquet and Triller, 2003; Dahan et al., 2003; Triller and Choquet, 2003; Charrier et al., 2006; Holcman and Triller, 2006; Ehlers et al., 2007). α7 nAChR (Buerli et al., 2010) and glycine receptors (Ehrensperger et al., 2007) display similar motional behavior: both exhibit high mobility in extrasynaptic areas and confined, low motion in peri-synaptic and synaptic domains. Confinement is inversely correlated to mobility (Meier et al., 2001; Ehlers et al., 2007; Buerli et al., 2010). In a recent study of inhibitory glycinergic receptors and their scaffolding anchorage protein at the postsynaptic density, gephyrin, PALM time-resolved superresolution imaging showed that gephyrin clusters are comprised of several sub-clusters, and that these undergo dynamic changes in the time-course of minutes (Specht et al., 2013). According to these authors, the morphological changes may correspond to the splitting and merging of gephyrin clusters in the postsynaptic density, whose size determines the number of receptors it can accommodate. Furthermore, the number of the two key inhibitory neurotransmitters – glycine and GABA_A_- increased with the number of gephyrin clusters at spinal cord synapses. This is another reflection of gephyrin’s ubiquity in inhibitory synapses: gephyrin is involved in the clustering of both glycine receptors and a major subset of GABA_A_ receptors; both compete for the same sites on the gephyrin molecule. Palmitoylation of Cys212 and Cys284 in gephyrin has recently been reported to be critical for the association of this protein with the postsynaptic membrane and also essential for its clustering (trimers, hexamers, and nonamers; Dejanovic et al., 2014). Lack of palmitoylation leads to mislocalization of gephyrin in non-synaptic regions. Conversely, increased palmitoylation is associated with gain-of-function, i.e., augmented inhibitory GABAergic transmission.

Interestingly, although the lifetime of the α7 nAChR in glutamatergic and GABAergic synapses was similar, the diffusion coefficient was faster in the inhibitory GABAergic peri-synaptic region, and a larger fraction of α7 nAChRs was found close to glutamatergic synapses. This latter may be related to the observation from the same authors that PICK1, a protein that regulates the trafficking of AMPA receptors (Hanley, 2008), also interacts with α7 nAChR, inhibiting its clustering (Baer et al., 2007). The peri-synaptic localization of α7 nAChRs on excitatory glutamatergic synapses may bear particular relevance during early postnatal development, when AMPA receptors are still absent from postsynaptic sites. During this period, and in particular during the first postnatal week (when their density is highest, even higher than in the adult), α7 nAChRs may be the effectors of LTP, either directly or in conjunction with NMDA receptors, and be able to depolarize dendritic spines, thus relieving voltage-dependent Mg^2+^ block mediated by NMDA receptors, and leading to synaptic plasticity (Fabian-Fine et al., 2001).

## CURRENT APPROACHES AND FUTURE PROSPECTS: ASSESSING NEUROTRANSMITTER RECEPTOR MOBILITY WITH NANOSCOPIC HIGH-TEMPORAL RESOLUTION

Fluorescence microscopy has recently experienced a series of interesting developments that have opened new avenues to study the fine structure and dynamics of synaptic constituents with unprecedented resolution (see recent reviews in Eggeling et al., 2013; Willig and Barrantes, 2014). Localization-based superresolution imaging techniques based on stochastic activation of photo-convertible (switchable) fluorescent probes have in theory inherently low temporal resolution because of the need to collect a considerable number of photons per molecule to accurately localize a given particle. The limitation is particularly apparent when investigating live-cell dynamics. However, the difficulty can be circumvented when studying highly dense collections of particles, and specific analytic techniques have been used to address this subject, as is the case with DAOSTORM –originally developed to study crowded stars in astronomy – (Holden et al., 2011) or CSSTORM (compressed sensing STORM; Zhu et al., 2012). In Neurobiology, these techniques are already proving fruitful for studying the mobility of synaptic molecules. A nanoscopic stochastic technique coined “universal point-accumulation-for-imaging-in-nanoscale-topography” (uPAINT; Giannone et al., 2010) can be used to render single-molecule diffusion maps at very high particle densities. Anti-R2 subunit of the glutamate receptor followed by ATTO^647N^ antibody labeling enabled these authors to map a high number of trajectories of AMPA receptors in a single dendritic spine. Another superresolution SPT technique, the combination of single-molecule stochastic nanoscopy (PALM, fPALM) with SPT (“sptPALM”), successfully used to follow the trajectories of membrane proteins (Manley et al., 2008) and optimized for measuring changes in dendritic spine morphology (Frost et al., 2012), was applied to Eos2-labeled glutamate subunit-1 of the AMPA receptor in hippocampal dendrites. AMPA-R accumulation was shown to arise from interactions of the receptor with the membrane rather than from clustering (Hoze et al., 2012). A recent comparison between uPAINT and sptPALM imaging of endogenous and overexpressed AMPA receptors, respectively, showed a remarkable agreement between the two techniques in reporting the number of receptors, confirmed with STED and electron microscopy. AMPA receptor nanodomains were also shown to change in shape in a highly dynamic fashion, often colocalizing with the scaffolding protein PSD95 (Nair et al., 2013). In the sptPALM study a massive amount of trajectories was analyzed, showing that AMPA receptors in hippocampal synapses are concentrated in nanoclusters of ∼70 nm containing about 20 receptor molecules each (Nair et al., 2013). Some clusters partially co-localize with the scaffolding protein PSD-95. As expected, AMPA receptors are retained transiently in the postsynaptic region and exhibit constrained mobility, whereas they are free to diffuse in extrasynaptic areas.

Another recent work using sptPALM was used to study the adenosine triphosphate (ATP)-gated P2X7 receptors, members of the purinergic receptor family, labeled with Dendra2 (Shrivastava et al., 2013). P2X7 receptors hardly diffuse in the synaptic region, and two populations of receptors were found in extra-synaptic regions: a rapidly diffusing population and one stabilized within nanoclusters of ∼100 nm diameter. Another important synaptic membrane protein has recently been studied with sptPALM: Calcium/calmodulin-dependent protein kinase II (CaMKII), an enzyme involved in synaptic plasticity and, indirectly, underlying memory formation. sptPALM was applied to rat hippocampal neurons to track single molecules of CaMKIIα (Lu et al., 2014). CaMKIIα exhibits at least three kinetic subpopulations, the major one regulated by actin dynamics, and enzyme mobility in spines was consistently slower than in dendrites, indicating the presence of physical obstacles or binding partners. Interestingly, NMDA-R stimulation triggered CaMKII activation and prompted the immobilization and presumably the binding of CaMKII in dendritic spines (Lu et al., 2014).

## CONCLUSION

Keeping synaptic strength at an adequate level is a functional requisite of both peripheral and central nervous system synapses, and it is the combination of the ± mechanisms outlined above that concertedly operate to maintain the functionally adequate density of neurotransmitter receptors. The mechanisms utilized by cells to achieve this equilibrium are complex, and vary between peripheral and CNS. A common feature is the transient immobilization of receptors in nanoscale compartments of the synapse as opposed to extrasynaptic regions, commonly achieved by clustering or by interaction with scaffolding non-receptor proteins. Our ability to interrogate the dynamics of receptors is currently limited to brief glimpses of the molecules’ entire lifetime, from synthesis to degradation, but nonetheless these snapshots provide useful hints about the organization and the functionally relevant spatiotemporal behavior of these important molecules in the synapse.

## Conflict of Interest Statement

The author declares that the research was conducted in the absence of any commercial or financial relationships that could be construed as a potential conflict of interest.

## ABBREVIATIONS

αBTX: α-bungarotoxin
CDx: methyl-β-cyclodextrin
FCS: fluorescence correlation spectroscopy
FRAP: fluorescence recovery after photobleaching
MSD: mean square displacement
nAChR: nicotinic acetylcholine receptor
SPT: single particle tracking
TIRF: total internal reflection fluorescence.
